# Supplementary material for: Does Motor Imagery Training Improve Service Performance in Tennis Players? A Systematic Review and Meta-Analysis
Source: Behav Sci (Basel). 2024 Mar 5;14(3):207. doi: 10.3390/bs14030207 (PMC10968362; doi:10.3390/bs14030207)
Supplement: Supplementary file 1 [file behavsci-14-00207-s001.zip › Table S1.pdf]

Table S1: Detailed search strategy.

Searched on December 6, 2023

**Databases**	**Search strategy**	**Results**
Web of Science (1964 – December 2023)	(AB=("motor imagery" OR "mental training" OR "movement imagery" OR "mental practice" OR "mental simulation" OR "cognitive training" OR "mental imagery" OR "mental rehearsal" OR "mental movements" OR "visual imagery")) AND AB=(tennis)	94
PubMed (1996 – December 2023)	("motor imagery"[Title/Abstract] OR "mental training"[Title/Abstract] OR "movement imagery"[Title/Abstract] OR "mental practice"[Title/Abstract] OR "mental simulation"[Title/Abstract] OR "cognitive training"[Title/Abstract] OR "mental imagery"[Title/Abstract] OR "mental rehearsal"[Title/Abstract] OR "mental movements"[Title/Abstract] OR "visual imagery"[Title/Abstract]) AND (tennis[Title/Abstract])	42
SPORTDiscus (1984 - December 2023)	AB ("motor imagery" OR "mental training" OR "movement imagery" OR "mental practice" OR "mental simulation" OR "cognitive training" OR "mental imagery" OR "mental rehearsal" OR "mental movements" OR "visual imagery") AND AB tennis	66
SCOPUS (2004 – December 2023)	TITLE-ABS-KEY ("motor imagery" OR "mental training" OR "movement imagery" OR "mental practice" OR "mental simulation" OR "cognitive training" OR "mental imagery" OR "mental rehearsal" OR "mental movements" OR "visual imagery") AND TITLE-ABS-KEY (tennis)	78
Total		280
