# Supplementary material for: Does Motor Imagery Training Improve Service Performance in Tennis Players? A Systematic Review and Meta-Analysis
Source: Behav Sci (Basel). 2024 Mar 5;14(3):207. doi: 10.3390/bs14030207 (PMC10968362; doi:10.3390/bs14030207)
Supplement: Supplementary file 1 [file behavsci-14-00207-s001.zip › Table S2.pdf]

Table S2 **The data used for meta-analyses**

Reference	Test	EG (pre-test)		EG (post-test)		N	CG (pre-test)		CG (post-test)		N
		Mean	SD	Mean	SD		Mean	SD	Mean	SD	
Atienza et al., 1998	Service accuracy	28.12	2.21	29.37	3.54	4	27.62	1.7	26.5	3.53	4
	Service speed	37.62	6.54	40.62	12.32	4	34.25	4.7	34.25	7.1	4
	Service technique	4.66	0.18	5.39	0.35	4	4.38	0.14	4.63	0.23	4
Robin et al., 2007 EG1	Service speed	146.2	5.5	146.6	6.7	10	145.1	5.8	144.4	6.1	5
	Service return accuracy	3.8	1.2	3.4	0.9	10	3.4	1.1	3.5	1	5
Robin et al., 2007 EG2	Service speed	144.7	5.7	166.1	6.4	10	145.1	5.8	144.4	6.1	5
	Service return accuracy	3.5	1.2	3.6	0.9	10	3.4	1.1	3.5	1	5
Coelho et al., 2007	Serve accuracy	10.8	2.2	12.3	2.1	23	10.4	1.9	10.3	1.9	25
	Service return accuracy	10.9	2.4	10.7	2.2	23	11.2	2.5	11.2	2.4	25
Guillot et al., 2012 EG1	Service speed	30.67	5.37	31.91	5.71	8	30.97	1.92	32.11	2.9	4
	Service return accuracy	12.38	4.03	10.63	4.84	8	7.71	5.35	6.57	3.1	4
Guillot et al., 2012 EG2	Service speed	34.32	6.41	36.34	5.82	7	30.97	1.92	32.11	2.9	3
	Serve accuracy	10.57	5.16	12.57	4.12	7	7.71	5.35	6.57	3.1	3
Dana et al., 2017 EG1	Service accuracy	183.91	38.5	73.83	21.3	12	195.25	34.9	134.83	28.1	6
Dana et al., 2017 EG2	Service accuracy	197.16	38.3	100.83	31.1	12	195.25	34.9	134.83	28.1	6
Fortes et al., 2019	Service accuracy	25.6	3.1	28.7	3.5	14	24.2	3.8	24.3	3.6	14
	Service speed	117.2	8	125.6	7.3	14	115.7	9.4	118.1	8.7	14
Robin et al., 2022 EG1	Service speed	145.7	5.7	150.2	3.9	11	143.3	4.5	146.6	5.2	6
	Service accuracy	41.79	33.43	55.19	18.94	11	43.20	33.43	48.66	20.06	6
Robin et al., 2022 EG2	Service speed	146.90	6.10	148.50	4.80	11	143.30	4.50	146.60	5.20	5
	Service accuracy	40.73	32.87	60.48	16.16	11	43.20	33.43	48.66	20.06	5

Robin et al., 2023 EG1	Service accuracy	1.91	0.67	5.18	1.11	11	2.27	1.14	2.55	1.44	6
	Service speed	80.57	34.12	92.55	29.45	11	55.71	23.63	64.64	20.64	6
	Service technique	2.41	0.37	2.90	0.39	11	2.06	0.44	2.12	0.35	6
Robin et al., 2023 EG2	Service accuracy	2.36	0.48	4.64	0.77	11	2.27	1.14	2.55	1.44	5
	Service speed	82.00	23.71	92.64	22.32	11	55.71	23.63	64.64	20.64	5
	Service technique	2.42	0.51	2.72	0.37	11	2.06	0.44	2.12	0.35	5
**Other data format**		**F**	**p**	**EG (N)**	**CG (N)**						
Fekih et al., 2020	Service speed	0.19	0.667	18	20						
	Service accuracy	10.52	0.003	18	20						

EG, experimental group; CG, control group
